# Bridging the Gap: A Synthetic X12 837i Claims Generator and Parser for Health Information and Informatics Education

**DOI:** 10.63116/ahisp-25-003

**Published:** 2026-04-17

**Authors:** Hants Williams

**Affiliations:** Stony Brook University

**Keywords:** health informatics education, synthetic data, X12 EDI, revenue cycle management, workforce readiness

## Abstract

**Background:**

Health information management education faces challenges in teaching claims data. Regulations prohibit the use of real, non-deidentified patient claims data within classroom settings. This limits hands-on experience with real-world data formats such as X12 837 institutional (837i) transaction files. Most existing approaches rely on textbook examples or static datasets that have been heavily redacted, which may lack the complexity required to prepare students for roles involving the handling of claims-related data. In this report, the authors describe the design and deployment of an open-source synthetic claims generator and parser designed to democratize access to realistic health care claims data, bridging the gap between theoretical knowledge and practical skills.

**Methods:**

A Python-based system was developed that generates realistic X12 formatted 837i claims using real Centers for Medicare & Medicaid Services (CMS) provider and payer databases. The tool features a free browser-based interface, plus local deployment via a command-line interface (CLI) or application programming interface (API) for advanced educational contexts. Finally, a modular two-lecture curriculum framework for integration into existing health information management courses is provided.

**Results:**

The browser-based application enables instant access without installation, eliminating technical and privacy barriers. The tool supports generating 1–25 claims per session via the web interface, with the ability to perform batch generation of thousands of claims via CLI/API for analytics projects locally on a user’s own machine. All generated files were validated against X12 5010 837i specifications using two independent electronic data interchange validation platforms, confirming zero structural errors and full conformance to industry standards. The framework aligns with American Health Information Management Association core competencies, offering a reproducible and scalable method to support workforce readiness for revenue cycle analyst, medical coding, and health care data analyst roles.

**Conclusions:**

By eliminating privacy concerns, financial barriers, and installation complexity, this method offers a scalable, reproducible model for teaching critical electronic data interchange and revenue cycle competencies related to the handling of claims data files. The open-source nature and multiple deployment options of this tool enable adoption across diverse institutional contexts, from community colleges to research universities, and from technical coding to nontechnical users.

## Introduction

Health information management (HIM) programs bear the responsibility of preparing students for a data-driven environment, where health care claims are the primary conduit to process roughly $4.9 trillion dollars worth of U.S. health care spending annually.^1^ The X12 data standard is used for electronic claim submission,^2^ and competency in this area is essential for roles including but not limited to revenue cycle management, analytics and data management, and population health.^3^^,^^4^ However, HIM- and health informatics-related educational programs face a critical instructional barrier: the inability to use real-world, raw claims data for hands-on training because of stringent patient privacy restrictions. Overcoming this barrier is essential to ensure that the next generation of HIM professionals develop the practical and technical proficiencies required to navigate this complex industry data standard effectively.

Health care privacy restrictions make it difficult for educators to train students on analyzing claims data that match real-world data structures.^5^ Educators therefore often rely on alternatives such as textbook examples, commercial electronic data interchange (EDI) tools, or synthetic data. Many commercial EDI tools do contain test data, but they present barriers beyond their typical high cost such as steep learning curves, lack of configurability or not customizable, and per-seat licensing restrictions that limit practical exploration and depth of training. Related to synthetic data, those datasets that are public domain, such as the public use files from Centers for Medicare & Medicaid Services (CMS),^6^ are not raw claim files but transformed into precleaned tabular data files. While synthetic data generation is growing across various healthcare domains, recent reviews indicate a lack of tools specifically tailored for X12 claims.^7^^,^^8^ Recent systematic reviews examining synthetic health data generation approaches^7^ and the domains were synthetic data is currently being produced^8^ health care, has identified no existing solutions specifically designed for synthetic X12 claims generation. Therefore, this article details the technical development and teaching implementation strategy for a newly developed open-source synthetic claims generator and parser specifically made for HIM education.

## The Innovation: A Multi-Interface Synthetic Claims Generator and Parser

### Generation of X12 837i Files

A synthetic claims generator was developed to produce high-fidelity X12 837 institutional (837i) transactional files in plain-text (.txt) format through structured logic. The system architecture consists of several interconnected components: (1) a generator and (2) a parser.

### Generator

The core generation engine is built in Python (3.8+) and integrates real-world reference data including CMS National Plan and Provider Enumeration System data for authentic provider National Provider Identifiers and taxonomies,^9^ a subset of approximately 14,000 International Classification of Diseases-10-CM diagnosis codes from official CMS release,^10^ publicly available Healthcare Common Procedure Coding System (HCPCS) procedure codes representing common facility services,^11^ 24,000-plus provider organizations with realistic practice locations,^12^ and the Healthcare.gov payer database with actual insurance product names and Health Insurance Oversight System (HIOS) IDs.^13^

Critically, although the generator leverages real-world reference data, no patient-level or real-world patient claims identifier data are ingested or used. All source data consist exclusively of publicly available, nonidentifiable code sets (diagnosis codes, procedure codes), provider directories (organizational National Provider Identifiers without patient linkage), and payer product catalogs. Patient identities, member IDs, claim numbers, service dates, and all transactional attributes are generated de novo using randomization algorithms with no relationship to actual health care encounters. This approach eliminates any risk of reidentification or data memorization, aligning with established frameworks for synthetic health data generation.^14^ The resulting files are structurally authentic but contain zero protected health information, making them suitable for unrestricted educational use.

**Figure 1. Example of Raw X12 837i Transaction Format Generated by the Synthetic Tool:**
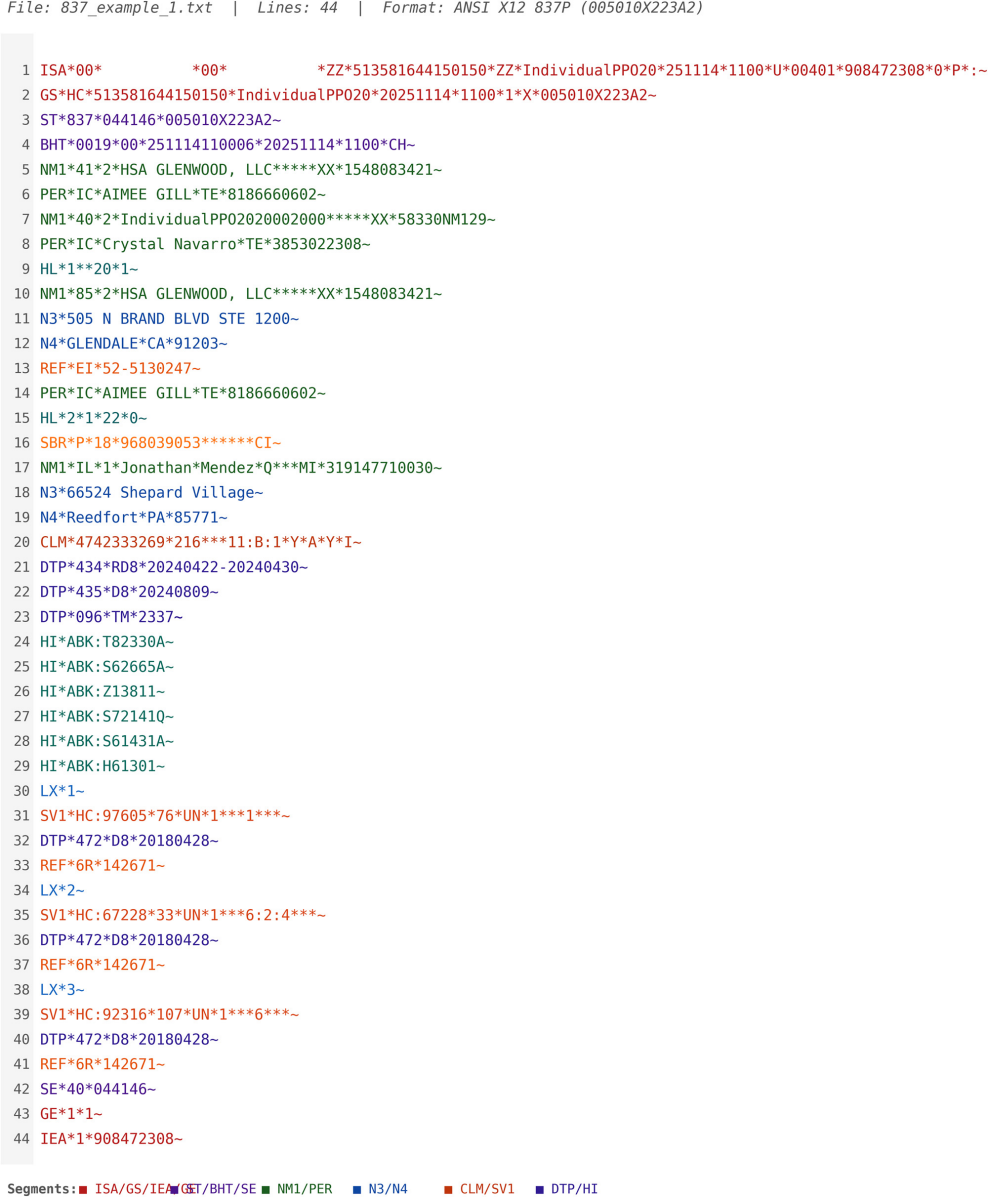


The generator maintains clinical and structural realism through proper X12 envelope structure (ISA, GS, ST segments), complete hierarchical loop formatting (2000A Billing Provider, 2000B Subscriber, 2300 Claim, 2400 Service Line), valid segment sequencing conforming to 5010 X12 implementation guides, diagnosis pointer relationships linking diagnoses to specific service lines, present-on-admission indicators for institutional quality reporting, and multiple service lines per claim (1–50 configurable) with proper line numbering. Figure 1 illustrates the raw X12 837i format generated by the tool, and Table 1 outlines the educational value associated with each generated component contained in the synthetic claim file.

**Table 1.** Educational Value and Competencies Associated With Synthetic Claim File Components

Component	Implementation Details	Educational Value
Clinical coding	Draws from 14,000+ ICD-10 diagnosis codes and publicly available HCPCS procedure codes; maintains clinical plausibility within broad categories	Students work with authentic medical code structures and relationships
Provider data	Integrates 24,000+ real NPIs from NPPES with associated taxonomies and practice locations	Claims contain realistic provider identifiers matching industry formats
Payer information	Uses actual insurance product names and HIOS IDs from Healthcare.gov database	Students see diverse payer types (Medicare, Medicaid, commercial) reflecting real markets
Structural hierarchy	Full X12 envelope with proper ISA/GS/ST headers; maintains 2000A/2000B/2300/2400 loop hierarchy	Students learn authentic EDI structure and segment sequencing rules
Validation logic	Configurable complexity (1–12 diagnoses, 1–50 service lines)	Enables scaffolded learning from simple to complex
Format validation	Generated files validated against X12 5010 837i specifications using external tools	Students work with production-grade EDI files that pass industry-standard validation

Abbreviations: EDI, electronic data interchange; HCPCS, Healthcare Common Procedure Coding System; HIOS, Health Insurance Oversight System; ICD, International Classification of Diseases; NPI, National Provider Identifier; NPPES, National Plan and Provider Enumeration System.

### Parser and Analytics Capabilities

Beyond 837i generation, the system also includes basic parsing capabilities that convert an X12-formatted 837i file into structured CSV files, addressing a major technical barrier for introductory students. The parser produces three separate outputs which include a header CSV containing transaction metadata (ISA sender/receiver, GS control numbers, ST transaction type, BHT reference data), a diagnoses CSV with diagnosis-level information (claim ID, diagnosis codes, diagnosis pointers), and a service lines CSV with service line details (procedure codes, charges, diagnosis pointers). This separation teaches students the relational structure of health care data while providing analytics-ready datasets that eliminate the need for custom parsing code. Table 2 shows the corresponding parsed CSV outputs, revealing how the parser transforms complex EDI syntax into analytics-ready relational data.

**Table 2:** Sample Header CSV Output Containing Transaction Metadata and Provider Identification

Column Name	Value
Transaction Set ID	837
Control Number	44146
Billing Provider	HSA GLENWOOD, LLC
Provider NPI	1548083421
Provider City	GLENDALE
Provider State	CA
Subscriber Name	Jonathan Mendez
Subscriber ID	3191511

Abbreviation: CSV, Comma-Separated Values.

**Table 3.** Sample Diagnoses CSV Output Illustrating Claim ID and Diagnosis Code Mapping

Claim ID	Diagnosis Type	Diagnosis Code	Pointer
4742333269	ABK	T82330A	1
4742333269	ABK	S62665A	2
4742333269	ABK	Z13811	3
4742333269	ABK	S72141Q	4
4742333269	ABK	S61431A	5
4742333269	ABK	H61301	6

Abbreviations: CSV, Comma-Separated Values; ABK, Principal Diagnosis.

**Table 4.** Sample Service Lines CSV Output With Procedure Codes and Diagnosis Pointers

Claim ID	Line #	Procedure Code	Charge	Unit	Qty	Diagnosis Pointers	Service Date
4742333269	1	HC:97605	76	UN	1	[1]	20180428
4742333269	2	HC:67228	33	UN	1	[2, 4, 6]	20180428
4742333269	3	HC:92316	107	UN	1	[6]	20180428

Abbreviation: CSV, Comma-Separated Values.

The relational structure shown in Tables 2, 3, and 4 mirrors real-world health care data architecture. Students learn to join tables by claim ID, understand diagnosis pointer relationships linking service lines to specific diagnoses, and perform analytics without writing custom parsing code. For example, service line 2 (procedure HC:67228) is linked to diagnoses 2, 4, and 6 via the diagnosis pointers, demonstrating how procedure codes must be justified by appropriate diagnoses for claim adjudication.

### Validation

To ensure structural and syntactic conformance to X12 5010 837i specifications, all generated claims were validated using two independent EDI validation platforms: DataInsight Health’s EDI Viewer^15^ and Stedi’s EDI Inspector.^16^ Both platforms parse X12 transaction files and identify segment-level errors, invalid loop structures, and conformance violations against the official 005010X223A2 implementation guide. Validation testing consisted of randomly generated claims with varying complexity parameters (1–12 diagnoses, 1–50 service lines, multiple provider types). All generated files successfully passed structural validation with zero segment sequencing errors, confirming proper ISA/GS/ST envelope structure, correct hierarchical loop nesting (2000A/2000B/2300/2400), and valid element separators and terminators.

## Deployment and Curriculum Integration

### Deployment Architecture

To accommodate varying student technical proficiency and pedagogical contexts, a multitier deployment strategy was implemented to maximize accessibility across differing levels of technical capabilities.

The first approach is a publicly available, browser-based web application. Using modern web technologies such as Pyodide^17^ and WebAssembly,^18^ a Python-based tool can be entirely deployed and accessed within a student's own web browser. This browser-based deployment with GitHub provides a zero-cost hosting solution that allows the tool to be accessible via the web,^19^ indefinitely for free on Microsoft infrastructure, requires no server-side processing with all computation happening client-side (in-browser) via Pyodide, and allows for continuous deployment of any updates or enhancements to the underlying code. This deployment approach eliminates traditional barriers by not requiring any software installation, works in any modern browser, and poses no privacy risk because the data are synthetic and never leave the student’s own device with their direct action. This means that students can generate claims data instantly from any computer, tablet, or phone without downloading programs.

The second approach provides a local (on-device) deployment option. For HIM programs that may require that students learn how to interact with the provided application programmable interface or command line interface directly on their own machine, or need institutional hosting, or want to customize the software, this can also be achieved. All the necessary code can be copied to the user’s own device, as well as a Docker^20^ containerization file that allows for single-command deployment. This approach opens the opportunity for institutions to perform their own branding and customization or integration with learning management systems such as Canvas, Blackboard, and Moodle. Finally, the on-premises deployment also enables high-volume batch generation or parsing processing, which is not available on the publicly available browser version.

### Curriculum Integration Framework

The tool is best paired with a modular, two-lecture unit that can be embedded into HIM courses such as data analytics or revenue cycle management or a health information system course. This approach divides the content into two distinct lectures: theoretical foundations and applied analytics. The initial lecture contextualizes the 837i transaction within the broader health care payment ecosystem, dissecting the structural rigidity of the X12 standard and the specific logic of segments. This prepares students for the subsequent applied laboratory phase, where they use the synthetic tool to generate, parse, and analyze claims data in a hands-on environment. Table 5 outlines specific learning objectives, active learning strategies, and assessment deliverables for the proposed theoretical and applied sessions. This approach is grounded in experiential learning theory^21^ and interprofessional education frameworks^22^ designed to bridge the critical gap between upstream medical coding and downstream billing. By moving beyond static textbook examples, students can engage in a near-authentic revenue cycle simulation that can mirror real-world quality assurance processes and directly supports American Health Information Management Association core competencies.^23^ This form of integration better prepares students to navigate complex claims data and address key workforce readiness needs for roles in revenue integrity and health care data analysis.

**Table 5.** A Two-Part Approach to Introducing HIM Students to X12: Theoretical Foundations and Applied Analytics

Session	Learning Objectives	Classroom Activity	Assessment
Lecture 1: Theoretical foundations	Identify core X12 control segments (ISA, GS, ST). Explain the 837i hierarchical loop structure (2000A-2400A). Map EDI data elements to real-world billing functions.	Instructor reviews raw EDI. Students manually annotate a segment string to isolate delimiters and identify the sender, receiver, and control numbers.	Segment identification quiz: Requires students to link X12 segment IDs (eg, claim, NM1) to their respective business data roles.
Lecture 2: Applied Analytics	Generate structurally valid synthetic 837i files. Deconstruct EDI files into relational CSV datasets. Validate clinical necessity using diagnosis pointer logic.	Students use tool to generate claims, parse them into CSVs, and cross-reference the Service Line and Diagnoses files to verify that procedures are linked to appropriate diagnoses via pointers.	Report: Students create a technical report identifying logic errors in generated claims.

Abbreviations: EDI, electronic data interchange; CSV, Comma-Separated Values; ISA, Interchange Control Header; GS, Functional Group Header; ST, Transaction Set Header.

## Discussion

The primary contribution of this work is the democratization of access to realistic 837i claim files for educational purposes. Historically, HIM education has been bifurcated between theoretical textbook resources and high-cost commercial solutions for claims training. By leveraging modern technologies to develop and deploy the 837i generator directly in the browser, this approach has removed many of the preexisting barriers. The approach described here shifts the computing burden from institutional servers to the client device and allows any student with a web browser to generate and analyze thousands of 837i claims without software installation, licensing fees, or privacy risks. This zero-cost, zero-infrastructure model creates an equitable pathway for institutions of all sizes to offer rigorous technical training. Beyond technical accessibility, this tool addresses a “skills gap” in revenue cycle management workforce preparation. Theoretical knowledge of the X12 standard is only a first step for roles in revenue integrity, claims analysis, and health data science. By providing students with 837i generation and parsing engines, we can better equip them with tangible exercises that allow them to improve their technical proficiency and workforce competitiveness for technical roles within HIM jobs.

Although this tool provides a high-fidelity simulation, it currently relies solely on synthetic logic, which lacks the erratic “messiness” of real-world human error found in production environments. In addition, the current iteration focuses exclusively on the institutional (837i) transaction set. Future development will prioritize the inclusion of the 837p (professional) transaction set to support ambulatory care curriculum, as well as the 835 (electronic remittance advice). Additional enhancements may include intentional error injection capabilities to simulate common coding mistakes and structural defects for quality assurance training, modeling longitudinal patient journeys to teach care continuity analysis, and incorporating more nuanced payer-specific adjudication logic. We invite HIM educators to contribute and partner with the open-source project. The web application is publicly accessible with source code and implementation guides available in the GitHub repository, which is permanently archived on Zenodo.^24^ Educators interested in collaborative research partnerships are encouraged to contact the authors.

## Conclusions

The synthetic X12 837i claims generator represents a practical, scalable solution to a persistent barrier in health informatics education. By eliminating privacy concerns, financial costs, and installation complexity, we have created a model that any institution can adopt regardless of resources or technical infrastructure.

## Acknowledgments

The authors thank and acknowledge Stony Brook University’s Applied Health Informatics Department with the School of Health Professions, as well as the open-source community for past and future contributions.

## Disclosures

The authors have nothing to disclose.

## Funding

The authors received no funding for this research.

## Data and Code Availability

The X12 837i synthetic claims generator is freely available as open-source software. The web application can be accessed at https://hantswilliams.github.io/x12-837-fake-data-generator. Source code, documentation, and deployment instructions are available on GitHub at https://github.com/hantswilliams/x12-837-fake-data-generator and permanently archived on Zenodo (https://doi.org/10.5281/zenodo.17611497). All code is released under the Creative Commons Attribution-Noncommercial 4.0 International License.

CE Quiz
